# Cannabis oil extracts for chronic pain: what else can be learned from another structured prospective cohort?

**DOI:** 10.1097/PR9.0000000000001143

**Published:** 2024-04-26

**Authors:** Dorit Pud, Suhail Aamar, Bareket Schiff-Keren, Roee Sheinfeld, Silviu Brill, Dror Robinson, Yaakov Fogelman, George Habib, Haggai Sharon, Howard Amital, Boris Boltyansky, Simon Haroutounian, Elon Eisenberg

**Affiliations:** a Faculty of Social Welfare and Health Sciences, University of Haifa, Haifa, Israel; b Hadassah-Hebrew University Medical Center, Jerusalem, Israel; c Schiff-Keren Pain Clinic, Tel-Aviv, Israel; d Institute for Pain Medicine, Chaim Sheba Medical Center, Tel Hashomer, Israel; e Pain Center, Tel Aviv Sourasky Medical Center, Tel Aviv, Israel; f Orthopedic Research Unit, Hasharon Hospital, Rabin Medical Center, Petah Tikwa, Israel; g Leumit Health Services, Tel Aviv, Israel; h The Ruth & Bruce Rappaport Faculty of Medicine, Technion-Israel Institute of Technology, Haifa, Israel; i Reumatology Unit, Laniado Hospital, Netanya, Israel; j Sagol Brain Institute and the Institute of Pain Medicine, Tel Aviv Medical Center, Tel Aviv, Israel; k Sackler Faculty of Medicine and Sagol School of Neuroscience, Tel Aviv, Israel; l Department of Anesthesiology, Washington University School of Medicine, St. Louis, MO, USA

**Keywords:** Chronic pain, Medical cannabis, Oil extract, Related symptoms

## Abstract

**Introduction:**

The use of medicinal cannabis for managing pain expands, although its efficacy and safety have not been fully established through randomized controlled trials.

**Objectives:**

This structured, prospective questionnaire-based cohort was aimed to assess long-term effectiveness and safety of cannabis oil extracts in patients with chronic pain.

**Methods:**

Adult Israeli patients licensed to use cannabis oil extracts for chronic pain were followed prospectively for 6 months. The primary outcome measure was change from baseline in average weekly pain intensity, and secondary outcomes were changes in related symptoms and quality of life, recorded before treatment initiation and 1, 3, and 6 months thereafter. Generalized linear mixed model was used to analyze changes over time. In addition, “responders” (≥30% reduction in weekly pain at any time point) were identified.

**Results:**

The study included 218 patients at baseline, and 188, 154, and 131 at 1, 3, and 6 months, respectively. At 6 months, the mean daily doses of cannabidiol and Δ9-tetrahydrocannabinol were 22.4 ± 24.0 mg and 20.8 ± 30.1 mg, respectively. Pain decreased from 7.9 ± 1.7 at baseline to 6.6 ± 2.2 at 6 months (*F*(3,450) = 26.22, *P* < 0.0001). Most secondary parameters also significantly improved. Of the 218 participants, 24% were “responders” but could not be identified by baseline parameters. “Responders” exhibited higher improvement in secondary outcomes. Adverse events were common but mostly nonserious.

**Conclusion:**

This prospective cohort demonstrated a modest overall long-term improvement in chronic pain and related symptoms and a reasonable safety profile with the use of relatively low doses of individually titrated Δ9-tetrahydrocannabinol and cannabidiol.

## Abstract 2

In real world, cannabis oil extracts containing relatively low Δ9-tetrahydrocannabinol and cannabidiol doses demonstrated improvement in pain and related symptoms, along with a fair safety profile.

Supplemental Digital Content is Available in the Text.

## 1. Introduction

Chronic pain is a significant challenge with substantial consequences for individuals and society as a whole.^10^ The available pain medications for chronic pain have limited effectiveness and often cause unfavorable side effects.^23^ Many clinical trials for new drug development have not achieved their primary goals, leaving the treatment of chronic pain as an unmet need. At the same time, a growing trend of using medicinal cannabis (MC), including cannabis-based medicinal products and herbal cannabis, for managing chronic pain is notable.

Although there are promising preclinical data supporting the potential analgesic efficacy of cannabinoids and modulators of the endocannabinoid system,^12^ there is a lack of high-quality evidence to conclusively support the clinical use of MC. Two recent systematic reviews and meta-analyses of randomized controlled trials (RCTs) on MC for chronic pain yielded mixed results. One review described the evidence as marginally effective,^30^ while the other review neither supported nor refuted the claims of efficacy and safety.^13^ Both reviews highlighted significant methodological flaws and a high or uncertain risk of bias in many of the included RCTs. The complexity of the cannabis plant, with its numerous active constituents beyond the major cannabinoids cannabidiol (CBD) and Δ9-tetrahydrocannabinol (THC), the various routes of administration (smoking, vaping, oral, sublingual, and topical), and the uncertainty surrounding dosing and titration regimens, all contribute to the difficulty in conducting successful RCTs with MC.

When RCTs fail to provide clinically useful information, the Grading of Recommendations Assessment, Development, and Evaluation (GRADE 2013)^15^ suggests turning to large observational studies as the next source of scientific knowledge. Several observational cohorts have been published in recent years, suggesting that MC may have a modest positive impact on chronic pain and related symptoms.^5^

Medicinal cannabis was first approved for compassionate use in Israel in 2005. Since then, the number of patients authorized by the Israeli Ministry of Health to use MC has exceeded 125,000, with chronic pain being the most common indication. The majority of patients consume MC by smoking or vaping the plant. Although at least 2 “real-life” prospective cohorts in Israel have studied the effectiveness and safety of MC, most patients in these studies consumed the plant itself.^3,16^

More recently, oil extracts of MC with standardized THC and CBD concentrations have become more readily available for sublingual use in Israel. However, there is still a lack of “real-life” data on daily dosages, titration, effectiveness, and safety of these compounds.

The objective of this current observational cohort study was to prospectively and systematically follow patients with chronic pain treated with oil extracts of MC. The study aimed to examine individual THC and CBD dosing and titration over a 6-month period to gain insights into real-life daily dosages of the major cannabinoids, effectiveness and safety of this route of administration, and search for associations between baseline measures and treatment outcomes.

## 2. Methods

### 2.1. Study design

This observational, prospective study was conducted from May 2019 to October 2021. The study protocol was registered at ClinicalTrials.gov (identifier: NCT04031313), after approval by the ethics committees of the University of Haifa (#216/19). Full trial protocol can be available upon request.

### 2.2. Participants and study conduct

Ten specialist physicians in Israel who routinely prescribe MC for the management of chronic pain collaborated on the study. The physicians (pain specialists, rheumatologists, or orthopedic surgeons) described this observational study procedures and obtained written informed consent from eligible participants. Copies of the consent forms along with the patients' pain diagnoses and contact information were sent to the study coordination center. Patients were contacted by an investigator and were asked to complete study questionnaires and information on MC dosing at baseline (before MC treatment initiation) and at 1, 3, and 6 months after MC treatment initiation. No financial compensation was offered to participating patients. To avoid any possible influence of collected data on physicians' decisions regarding clinical management of their patients, prescribing physicians had no access to data collected on individual patients.

Eligible patients were selected by the collaborating physicians according to their own clinical judgement only, as long as they were Hebrew-speaking, age 18 years and older applying for a first-time MC license for treating any form of chronic noncancer-related pain.

### 2.3. Study questionnaires

Data were collected online by the secured survey technology Qualtrics (version 12018, 2015; Qualtrics, Provo, UT).

Physicians reported data on pain etiology using the International Classification of Diseases-11 code. Baseline patient questionnaires included information on age, sex, body mass index (BMI), pain diagnosis, comorbidities, and level of expectation from treatment's success (0-10 scale). The following data were collected at baseline and at the 3 follow-up time points: average weekly pain intensity and average daily pain intensity (Numeric Pain Scale, 0-10, primary outcome); THC/CBD consumption (milligrams per day); opioid consumption (yes/no); 7 Hebrew validated versions of the following questionnaires (secondary outcomes): short-form McGill Pain Questionnaire^22^; Pain Disability Index^26^; quality of life, EuroQol^8^; Pittsburgh Sleep Quality Index^9^; Beck Depression Inventory II^4^; General Anxiety Disorder^28^; and Pain Catastrophizing Scale.^29^ Also, patients were requested to report adverse events (AEs) at each follow-up time-point. A detailed list of potential AEs was made available to patients who were requested to check if they have experienced any of them. AEs were later classified as serious or nonserious, according to the Food and Drug Administration definition.^24^

### 2.4. Consumed cannabinoids

The use of MC in Israel requires licensing from the Ministry of Health and prescriptions for 1 or more prefixed combinations of THC:CBD oil extracts (T20:C4 [=20% THC:4%CBD]; T15:C3; T10:C2; T10:C10; T5:C5; T5:C10; T3:C15; T1:C20; T0:C24), provided by several different manufacturers. The THC:CBD extracts are obtained by the patients with a prescription, from a licensed pharmacy. Notably, the administration of THC alone is not possible, so titration of each component by itself is somewhat limited. The decision on dose, combination, and manufacturer was made by the prescribing physician and was unrelated to the conduct of this study.

### 2.5. Statistical analysis

Patients who completed the baseline study questionnaire and received at least 1 dose of MC were qualified for analysis. Procedure GLIMMIX by SAS software (version 9.4) was used to analyze changes over time of each outcome measure by generalized linear mixed model. The model was defined by random intercepts only. The model was tested several times while including many potential confounding factors such as BMI, age, sex, pain type (ie, nociceptive, neuropathic, nociplastic, visceral, and headache), use of opioids, comorbidities, and CBD and THC doses. Of all those factors, age and sex were found significant. In an attempt to be as parsimony as possible, nonsignificant factors were excluded. Therefore, the final model was adjusted for sex and age only. The assumption of the normal data distribution in the generalized linear mixed model was tested by the distribution of the residuals. This assumption of normality was met and values are therefore presented as mean and SD. Adverse events were grouped by their occurrence (yes/no) in different body systems (eg, central nervous system [CNS], gastrointestinal [GI]) and their probability of occurrence was tested by using a logistic mixed model, in which CBD and THC doses and time points were entered as potential predictors.

Because of the prospective, longitudinal data collection design, each of the time points had a different sample size, which was analyzed with the corresponding baseline information.

An additional analysis was aimed to classify the patients as “responders” (those who reported 30% or more reduction from baseline in their average weekly pain at either 1, 3, or 6 months) and “nonresponders” (patients who failed to achieve this threshold) and to identify baseline factors that could distinguish between the 2 subgroups, including sex, age, BMI, pain diagnosis, and all baseline questionnaires scores. *T* tests were performed for comparisons between subgroups. In addition, Cohen's *d* tests were conducted for calculating the effect size of each outcome measure at each time point. Differences were considered significant at the *P* ≤ 0.05 level.

## 3. Results

### 3.1. Demographic and clinical characteristics

**Figure 1.**
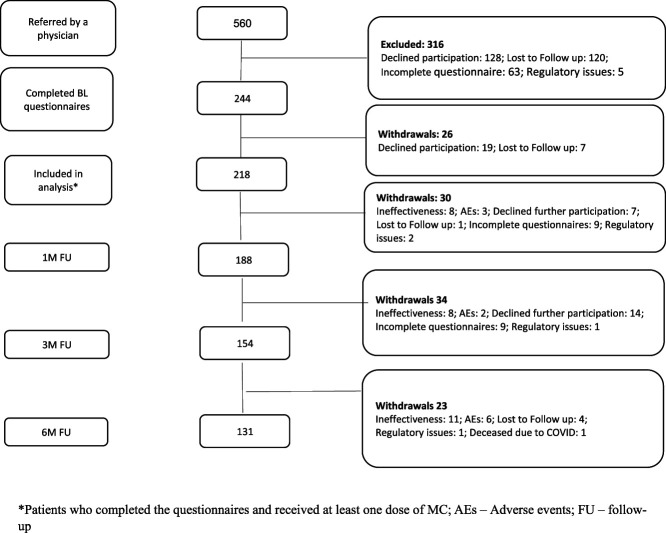
Patient flow diagram.

According to the Israeli Ministry of Health regulations, all patients were treated by a specialist for their pain diagnosis for at least 1 year and failed to obtain satisfactory pain relief, before initiation of MC. A total of 218 patients were eligible for the study. Their mean age was 54 ± 15 years, and 77% (n = 168) were female. Mean BMI was 27 ± 5.9. Nearly 80% of this sample reported at least 1 additional comorbidity. The primary pain diagnosis was nociplastic pain (98, 44%), followed by nociceptive (55, 25%), neuropathic (48, 21%), headache (13, 6%), and visceral pain (9, 4%). Level of expectation for treatment's success on a 0 to 10 scale was 8.9 ± 2.3. Of the 218 eligible patients at baseline, 188, 154, and 131 patients at 1, 3, and 6 months, respectively, were qualified for this study analysis (Fig. 1).

### 3.2. Cannabinoids

Mean THC daily dose gradually increased from 13.2 ± 15.3 mg/d at 1 month to 17.2 ± 18.2 mg/d at 3 months and to 20.8 ± 30.1 mg/d at 6- months. The mean CBD daily dose remained relatively stable: 20.1 ± 23.2, 24.1 ± 24.6, and 22.4 ± 24.0 mg/d at 1, 3, and 6 months, respectively.

### 3.3. Effect on pain and accompanied symptoms

The mixed-model analysis (while adjusting for age and sex) revealed a statistically significant improvement from baseline in all outcome measures at all 3 time points, except for depression (Table 1).

**Table 1:** Outcome measures over time.

	Baseline (n = 218) Mean ± SD	1 mo (n = 188) Mean ± SD	3 mo (n = 154) Mean ± SD	6 mo (n = 131) Mean ± SD	Mixed model analysis
Weekly pain	7.9 ± 1.7	7.0 ± 2.1	6.8 ± 2.3	6.6 ± 2.2	*F*(3,450) = 26.22, *P* < 0.0001
Daily pain	7.6 ± 1.89	6.7 ± 2.2	6.4 ± 2.4	6.2 ± 2.5	*F*(3,445) = 20.46, *P* < 0.0001
McGill total	23.5 ± 10.7	20.5 ± 10.8	21.2 ± 10.6	21.0 ± 10.5	*F*(3,458) = 8.57, *P* < 0.0001
McGill sensory	17.8 ± 8.0	16.1 ± 8.1	16.2 ± 8.0	16.1 ± 8.0	*F*(3,454) = 6.0177, *P* = 0.0005
McGill affective	6.2 ± 3.0	5.2 ± 3.0	5.4 ± 3.2	5.2 ± 3.0	*F*(3,415) = 11.83, *P* < 0.0001
Sleep	12.3 ± 4.2	10.3 ± 4.2	10.3 ± 3.7	10.9 ± 4.0	*F*(3,455) = 24.81, *P* < 0.0001
Pain catastrophizing	32.3 ± 11.4	30.2 ± 13.1	27.1 ± 13.2	26.2 ± 12.8	*F*(3,441) = 17.89, *P* < 0.0001
Anxiety	8.4 ± 6.2	7.1 ± 5.9	7.0 ± 5.9	6.5 ± 5.7	*F*(3,441) = 10.87, *P* < 0.0001
Depression	8.2 ± 8.1	7.9 ± 6.6	7.4 ± 6.8	7.5 ± 8.1	*F*(3,170) = 0.8, *P* = 0.5
Disability	6.1 ± 2.1	5.4 ± 2.1	5.3 ± 2.3	4.9 ± 2.2	*F*(3,433) = 23.54, *P* < 0.0001
Quality of life	4.2 ± 1.8	3.8 ± 1.7	3.7 ± 1.8	3.6 ± 1.6	*F*(3,432) = 14.16, *P* < 0.0001

When transferred to percentage of change from baseline, maximal improvement in all outcome measures was noted at the 6-month time point. Thus, average weekly pain decreased by 14%, average daily pain by 12%, anxiety by 9%, in pain catastrophizing by 16%, quality of life impairment by 12%, and disability by 15%. Sleep disturbance maximally improved by 10%, but at the 3-month follow-up. The number of patients consuming opioids also decreased from 43 at baseline to 9, 10, and 9 at the 3 follow-up time points.

Of the 218 baseline participants, 24% (n = 52) reported 30% or more reduction from baseline in their average weekly pain at least at 1 follow-up time point and were defined as responders. However, none of the baseline factors (sex, age, BMI, pain diagnosis, and baseline questionnaires scores) could distinguish the responders from the nonresponders. The only exception was pain catastrophizing, which was significantly higher at baseline among the responders (score of 35 vs 31; *P* < 0.046) (Supplemental Table 1, available at http://links.lww.com/PR9/A222). Among the responders, other outcome measures such as sleep, disability, and quality of life also showed higher magnitudes of improvement (Supplemental Table 2, available at http://links.lww.com/PR9/A222). Markedly, 80% of the responders completed the 6-month follow-up, in contrast to only 55% of the nonresponders.

### 3.4. Adverse events

Up to 52% of patients reported AEs at the 1-month time point, and less frequently so thereafter. Most AEs were graded as nonserious^24^ and, according to the affected system, were most commonly related to the CNS followed by the GI system (Table 2).

**Table 2:** Adverse events according to affected system at the different time points.

System	1 mo n (%)	3 mo n (%)	6 mo n (%)
General (any)	99 (52)	78 (51)	42 (32)
Central nervous system	64 (34)	39 (25)	22 (17)
Gastrointestinal	35 (19)	29 (19)	12 (9)
Psychological	30 (16)	25 (16)	13 (10)
Musculoskeletal	22 (12)	30 (19)	9 (7)
Cardiovascular	12 (6)	10 (6)	6 (5)
Visual	19 (10)	20 (13)	7 (5)
Auditory	12 (6)	5 (3)	3 (2)

Overall, 11 patients discontinued the study due to AEs, although we cannot rule out the possibility that additional patients who declined further participation have not completed the questionnaires or were lost to follow-up opted to do so due to AEs.

According to the mixed-model analysis, presence of AEs was not explained by time elapsed since treatment initiation nor by THC or CBD doses. A detailed report of all AEs can be found in Supplemental Table 3 (available at http://links.lww.com/PR9/A222).

Serious AEs (SAEs) requiring inpatient hospitalization^24^ were reported by 9 patients: 3 patients in relation to cardiac AEs, 3 due to GI, 2 psychiatric, and 1 due to CNS problems. In 6 of them, hospitalization took place during the first month of treatment. In the other 3, hospitalization was reported at the 6-month time point and no follow-up was possible. Notably, 1 additional patient passed away due to COVID-19 disease, likely unrelated to MC treatment.

## 4. Discussion

This prospective cohort of a considerably large population of patients with chronic pain presents data on the effectiveness and safety of oil extracts of MC. With respect to effectiveness, the mixed-model analysis demonstrated significant improvement in the primary outcome, which is the change from baseline in average weekly pain intensity, at all 3 time points. The maximal reduction in pain from 7.9 ± 1.7 at baseline to 6.6 ± 2.2 was noted at the 6-month time point and was equivalent to a 14% reduction. When looking at a subgroup of patients who achieved a 30% or more reduction in pain from baseline, 24% were defined, accordingly, as responders. Several other cohorts of patients with chronic pain treated with cannabis have been published in recent years, so comparing the results may have an added value in terms of validating MC effectiveness. For example, the UK Medical Cannabis Registry of patients who received full-spectrum cannabis oil extracts found a similar magnitude of reduction in the visual analogue scale, from 6.3 ± 1.7 to 5.4 ± 2.5 at 6 months, but failed to reach statistical significance most likely because of the small number of patients (n = 12) who reached that time point.^19^ In another cohort of 206 patients who were treated mostly by full-spectrum inflorescence (but some by oil extracts), average pain severity score dropped from 7.50 (95% confidence interval [CI], 6.75-7.75) to 6.25 (95% CI, 5.75-6.75) at 6 months.^16^ Yet, another cohort of 851 patients treated mostly by cannabis inflorescence demonstrated roughly 20% reduction in pain from baseline at 6 months.^3^ Finally, a recent meta-analysis and systematic review of 6 cohort studies with 2571 patients found a weighted mean difference of mean pain reduction of 1.75 (95% CI, 0.72-2.78) on a 0 to 10 scale.^5^ Thus, one may conclude that “in real life,” cannabis produces a modest analgesic effect, regardless of its administration route. Whether or not this effect is within or beyond the expected magnitude of placebo analgesia has recently been under debate.^1,17,21^

Doses of the cannabis major constituents' THC and CBD vary considerably between the cohorts. In the Haroutounian study, for example,^16^ mean calculated cannabis daily THC dosage used (primarily by inflorescence) was 144 mg, which is 7 times higher than the dose used in the current cohort. Similar magnitude of high doses was consumed in Aviram's study, again primarily by inflorescence.^3^ By contrast, in a retrospective cohort of Danish patients,^17^ median daily CBD/THC oil extract doses ranged between 7.9 and 13.2 mg, which is much closer to the range used by our patients, and well within a recent consensus-based recommended range.^6^ Taken together, these observational cohorts suggest that in practice, much lower doses (at a range of 1 order) of oil extracts of cannabis are used compared with inflorescence (in other words, smoking or vaping it). It therefore seems that oil extracts may be advantageous over inflorescence as they allow more precise dosing, lower THC dose consumption, and comparable analgesia.

The present cohort also emphasizes the effect of cannabis on many of the other symptoms, which are often reported by patients with chronic pain—namely anxiety, impaired sleep, depression, and catastrophizing. It also has a positive effect on functioning and health-related quality of life. All these effects are modest in size but are rather consistent and congruent with those found in additional cohorts.^5^ Hence, cannabis seems to have an impact on the “disease burden” of chronic pain rather than being a potent analgesic per se. We suggest to take this into consideration in future studies on cannabis for chronic pain.

Two additional points deserve consideration regarding the effectiveness of cannabis use: First, in the present cohort, the number of patients consuming opioids decreased over time, in line with at least 1 other report.^3^ However, because only a minority of participants in the present cohort consumed opioids at baseline and only at low doses, we wish to avoid drawing conclusions about an opioid-sparing effect of cannabis use.

Second, sex and age were found as confounders and the statistical models were therefore adjusted accordingly. Sex and age differences in response to analgesia in patients with chronic pain have been widely reported.^11^ At the same time, evidence regarding sex and age differences in response to cannabis analgesia is still limited and equivocal.^7,20^ Thus, determining whether these confounding effects are specific to cannabis or are inherent to analgesia in general is challenging.

Retrospective subgrouping of patients (ie, “responders” vs “nonresponders”) has been suggested as an elegant statistical method for identifying factors contributing to a treatment response.^25^ Accordingly, we retrospectively classified our patients into “responders” and “nonresponders” but unfortunately failed to identify neither objective nor patient-reported baseline characteristics that may predict treatment success (except for catastrophizing). This somewhat contradicts the finding of another cannabis cohort, which was able to point predictors for good response (≥30% decrease in average pain intensity) including normal to long sleep duration, lower BMI, lower depression scores, and a diagnosis other than neuropathic pain.^3^ Despite these contradictory findings regarding predictors of response, it is noteworthy that the responding patients exhibited larger improvement in all other outcome measures relative to the “nonresponders,” pointing again to the effectiveness for the “disease burden.” A second noteworthy point is the lack of difference between the 2 subgroups in the expectation level at baseline regarding treatment's success. This may reduce the likelihood of attributing the observed effects entirely to placebo, because placebo is closely related to expectations.^2^

Safety continues to be a major concern regarding the medicinal use of cannabis. Overall, up to 45% of our patients reported any AE at any time point during the study, more commonly at the first month of treatment. Other surveys reported a similar range of AEs: 30%,^18^ one-third of patients,^14^ and 30% to 40%.^3^ Most AEs are typically mild to moderate and allow continuous use of cannabis. Nine of our patients required hospitalization (4.5% of eligible patients at baseline) and are therefore considered as having SAEs. Because of the nature of this study, which relied on subjective, often questionnaire-based patient reports, no formal medical information could be obtained. Therefore, the relationship of the hospitalizations to the cannabis use remains questionable, although cannot be completely excluded. Nonetheless, attention should be given to possible associations between cannabis use and cardiovascular events^27^ and severe psychiatric illness.^18^

Several limitations of this study should be acknowledged. First, there may have been self-report bias, which was mitigated by using validated questionnaires and maintaining patient anonymity from their physicians. Second, lack of controls and dropout rates are inherent limitations of cohort studies like this one. This was best handled by using the mixed-model analysis. Third, the potential confounding effect of additional treatments such as surgery, physiotherapy, and alternative medicine was not collected and analyzed in the model.

In conclusion, this structured, prospective cohort study demonstrated modest improvements in pain, associated symptoms, functioning, and quality of life, and a reduction in opioid use. The reduction in “disease burden” was more pronounced in nearly a quarter of the patients, but no predictors for treatment success could be identified before treatment initiation. The doses of THC and CBD in the oil extracts were modest and considerably lower than those required to achieve similar magnitude of effect by cannabis inflorescence. Although medical cannabis treatment appears to be generally safe for most patients, some still experience SAEs.

## Disclosures

D. Pud received research grants from the Israel Pain Association and from Rafa Laboratories; H. Sharon received research grants, consulting or speaking fees, and/or honoraria from the Israel Pain Association, Bioevents, and Cellen Health; S. Haroutounian received research support or consulting fees from Disarm Therapeutics, Rafa Laboratories, GW Pharma, and Vertex Pharmaceuticals; E. Eisenberg received research grants, consulting or speaking fees, and/or honoraria from the Israel Pain Association, Rafa Laboratories, Syqe Medical, Teva Israel, Pfizer, Little Green Pharma, Bioevents, Greenwich Biosciences, Vectura Ferin Pharma, and Cannabotech. The other authors have no conflict of interest to declare.

## Appendix A. Supplemental digital content

Supplemental digital content associated with this article can be found online at http://links.lww.com/PR9/A222.
